# 80. Macrophage myofasciitis: a rare cause of severe arthro-myalgias

**DOI:** 10.1093/rap/rky034.043

**Published:** 2018-09-20

**Authors:** Shabnam S Shabbir, Maxton Pitcher, Dhiren Shah, Shahir S Hamdulay

**Affiliations:** 1 Rheumatology, North West Thames, London, UNITED KINGDOM; 2 Gastroenterology, Northwick Park Hospital, London, UNITED KINGDOM; 3 Radiology, Northwick Park Hospital, London, UNITED KINGDOM; 4 Rheumatology, Northwick Park Hospital, London, UNITED KINGDOM

**Introduction:** Macrophage myofasciits is a rare cause of muscle pain related to aluminium hydroxide following intramuscular vaccinations. Small patient cohorts and case reports associate the condition with classical PAS-positive macrophages on biopsy. Here we present a challenging case of macrophage myofasciitis which was PAS-negative on biopsy and refractory to steroid reduction. The patient is now established on reducing oral corticosteroids and subcutaneous methotrexate.

**Case description:** A 63 year old retired commercial airline attendant presented in December 2017 with an abrupt onset of severe pain and stiffness in the calves, thighs, arms and shoulders with fevers and night sweats. There was no reported infectious prodrome. Past medical history included hypertension, hypercholesterolaemia, left shoulder arthroscopy and cholecystectomy. Regular medications included ramipril, bendroflumethiazide, atorvastatin, omperazole, ibuprofen and paracetamol. He had received multiple travel vaccines over several decades. Clinical examination on presentation revealed stiffness on mobilising and tender biceps, calves and thighs. Neurological examination was unremarkable. There was no rash, lymphadenopathy or peripheral synovitis. Cardiovascular, respiratory and abdominal examinations were unremarkable. Blood tests on presentation showed a CRP 85, ESR 50 and WBC 15 (neutrophilia). A working diagnosis of polymyalgia rheumatica was proposed with the need to exclude malignancy and occult infection. Cultures for blood and urine were negative. TFT’s, PSA and urine EPS were negative. The patient was commenced on prednisolone 20mg once daily. One week after starting steroids there was a deterioration in symptoms with worsening muscle pains, fever, irritability, insomnia nocturnal pain and pleuritic chest pains. Blood testing revealed a further deterioration in acute phase markers (peak WCC 34, CRP 350 and ESR 85), with the presence of a hepatitis (peak ALT 180 , ALP 230 and GGT 600), hyperferritinemia (2600) and low albumin (22g/dl). CK levels remained normal and autoantibody testing was negative (ANA, ENA, RhF, anti-CCP, myositis panel, HMG CoA reductase ab negative). The patient had severe pain requiring high dose opioid analgesia (morphine), NSAIDs (celecoxib), IV paracetamol and nefopam to control pain.

MRI of the thighs revealed bilateral myofascial oedema affecting the quadriceps and hamstring muscles. CT PET revealed high signal from the fascial areas. A subsequent myofascial muscle biopsy showed PAS negative macrophagic infiltration in the fascia and muscles. MRCP and ECHO were within normal limits. EMG showed myopathic changes alone. A diagnosis of macrophage myofasciitis was made and the patient was treated with high dose prednisolone (60mg once daily). Over the following two weeks pain and stiffness slowly improved, which correlated with an improvement in inflammatory markers and liver function tests. Unfortunately prednisolone resulted in mood lability and insomnia. Analgesics were weaned to cessation and the patient was commenced on mycophenolate as a second line agent prior to discharge. Despite mycophenolate 1.25g twice daily, there was a relapse of limb pain, stiffness and inflammatory markers (CRP 55, WBC 15) on decreasing prednisolone to 30mg once daily. As liver function tests were near normal, mycophenolate was switched to subcutaneous methotrexate (20mg weekly) and prednisolone was successfully weaned to 10mg once daily. This resulted in normalisation of the inflammatory markers (CRP 7, ESR 10 and WBC 11) and resolution of pain and stiffness.

**Discussion:** Macrophage myofasciitis is a rare inflammatory syndrome which typically affects middle aged adults and is characterised by the presence of aluminium hydroxide (Alum) crystals within macrophages at sites of previous intramuscular vaccinations, classically the left deltoid. Clinical presentation may occur up to 10 years after vaccination and includes arthro-myalgia, chronic fatigue and cognitive impairment. A study of 130 patients with a similar presentation and history of aluminium-containing vaccines showed that one third had biopsy proven macrophage myofasciitis. It is considered part of the autoimmune/inflammatory syndrome induced adjuvants or ASIA spectrum of diseases. It has been associated with an increase in cytokines CCL2/MCP-1 levels compared to healthy subjects. Histopathology characteristically shows focal epi-, peri- and endomysial inflammatory infiltrate with PAS-positive macrophages and CD8+ T-cells. There is no significant muscle fibre injury. This case aims to raise awareness of this disease which must be included in the differential of patients presenting with inflammatory arthro-myalgias such as polymyalgia rheumatica and inflammatory myositis. MRI and PET-CT with targeted biopsy will lead to the diagnosis and methotrexate appears to be the DMARD of choice.

**Key Learning Points:** Consider macrophage myofasciitis in cases of resistant PMR and inflammatory pain. MRI and PET-CT useful in establishing diagnosis with myofascial biopsy. Methotrexate is the drug of choice in this single case.

**Disclosure: S.S. Shabbir:** None. **M. Pitcher:** None. **D. Shah:** None. **S.S. Hamdulay:** None.

